# The Antidiabetic Effect of Grape Skin Extracts of Selected Indigenous Croatian White Grapevine Varieties

**DOI:** 10.3390/foods13244143

**Published:** 2024-12-20

**Authors:** Vesna Rastija, Mato Drenjančević, Toni Kujundžić, Luka Zmaić, Maja Karnaš

**Affiliations:** 1 Faculty of Agrobiotechnical Sciences Osijek, Josip Juraj Strossmayer University of Osijek, Vladimira Preloga 1, HR-31000 Osijek, Croatia; vrastija@fazos.hr (V.R.); mato.drenjancevic@fazos.hr (M.D.); toni.kujundzic@fazos.hr (T.K.); 2 Faculty of Medicine Osijek, Josip Juraj Strossmayer University of Osijek, HR-31000 Osijek, Croatia; lzmaic@mefos.hr

**Keywords:** indigenous Croatian grape varieties, grape skin, antidiabetic potential, inhibition, α-amylase, α-glucosidase, antioxidant activity

## Abstract

Grape skin is an excellent bioactive compound source with numerous beneficial health effects. This study aimed to determine and compare the antidiabetic potential of the grape skin of indigenous Croatian white grapevine varieties. The grape skin extracts (GSEs) were assessed for total polyphenols, antioxidant activity, and inhibition potential against α-amylase and α-glucosidase, enzymes responsible for carbohydrate metabolism. GSE of variety “Svetokriška belina” has the highest total phenols (TP) content (1404.87 mg of gallic acid equivalent), the highest antioxidant capacity against DPPH (544.82 mg ascorbic acid equivalent), and the highest inhibitory activity against α-amylase (99.60%). α-glucosidase was best inhibited by the variety “Kozjak” (93.53%), followed by a significantly lower inhibition by the GSE of “Svetokriška belina” (89.64%). The principal component analysis (PCA) revealed the relationship among the grape varieties by their inhibition potential, where the first PC explained 71.34% of the variation. Indigenous Croatian white grapevine varieties have great potential for developing new natural supplements to prevent and treat diabetes.

## 1. Introduction

Diabetes mellitus (DM) is a chronic metabolic disorder characterized by chronic hyperglycemia due to disturbances of carbohydrate metabolism resulting from defects in insulin secretion, insulin action, or both. Type 2 diabetes mellitus (T2DM) is a common major form of diabetes that occurs when insulin resistance or deficiency leads to high blood glucose characterized by hyperglycemia. Untreated type 2 diabetes is associated with an array of retinal, renal, and neuropathic complications, as well as coronary artery and peripheral vascular disease [1].

One therapeutic approach for treating T2DM is lowering blood glucose levels by inhibiting the carbohydrate-hydrolyzing enzymes α-amylase (AA) and α-glucosidase (AG). Both enzymes, responsible for the breakdown of oligosaccharides and disaccharides into monosaccharides suitable for absorption, are present in the small intestinal brush border. Inhibitors of AA and AG are clinically approved drugs for the prevention and treatment of diabetes. Drugs such as acarbose, voglibose, and miglitol prevent the degradation of complex carbohydrates into glucose and reduce postprandial blood glucose levels [2,3]. However, synthetic drugs have numerous side effects, especially related to the gastrointestinal tract, (flatulence, diarrhea, vomiting, and abdominal pain), as well as hypoglycemia, headache, cardiovascular disease, and hepatotoxic potential [4,5]. Therefore, there is an increasing interest in discovering effective new inhibitors of AA and AG from diverse natural sources (food herbs and medicinal plants) to reduce the side effects of antidiabetic drugs. Most of these inhibitors are pregnanes, alkaloids, terpenes, polyphenols, and carotenoids [6,7,8,9]. For instance, four steroidal pregnane phytochemicals derived from the plant *Gongronema latifolium* showed greater inhibitory potential against porcine pancreatic alpha-amylase in vitro than the standard inhibitor, acarbose [6]. Terpenes isolated from *Capsella bursa-pastoris* Linn. shown inhibitory potential towards AA and AG [10]. The extracts of plants, *Sclerocarya birrea* and *Ziziphus mucronata*, as well as traditional medicinal plants (sweet gale (*Myrica gale* L.), roseroot (*Rhodiola rosea* L.), sheep sorrel (*Rumex acetosa* L.), stinging nettles (*Utrica dioica* L.), and dandelion (*Taraxacum officinale* L.)) demonstrated better AA and AG inhibitory activity than acarbose, attributed to the high content of polyphenols [11,12].

Grape skin is an excellent source of natural bioactive compounds such as stilbenes, polyphenols (phenolic acids, flavonoids, and tannins), fatty acids, higher alcohols, sterols, and terpenes/terpenoids [13,14,15,16]. Numerous beneficial health effects of grape skin extracts have been reported, such as antioxidant activity [14,15,16,17], anticancer activity [18,19], antimicrobial activity [20], and antidiabetic effects by the trapping of reactive carbonyl species [21] or the inhibition of AA and AG [22,23,24]. With white grape skin not being regularly used in wine production, it presents an interesting and valuable material for the production of health-promoting dietary supplements.

Many grape varieties are considered indigenous to the Republic of Croatia, as they originated and have been cultivated for hundreds of years in the same region. These varieties are essential for preserving the country’s biodiversity and cultural heritage. They are well adapted to the specific agroecological conditions of Croatian wine regions, and the wines made from these grapes suit the tastes and preferences of local consumers. Today, these varieties are the pillars of Croatian wine production specificity on the world market [25].

Additionally, these varieties play a crucial role in tourism by attracting visitors seeking authentic experiences tied to a specific area, and their protection helps safeguard the traditions and identity of Croatian winemakers [26]. As a result of research confirming the health benefits of these grapes and wines, they can be positioned as natural sources of beneficial compounds [27]. This type of branding could appeal to consumers looking for health-enhancing food products. Educating consumers about these health benefits can help indigenous varieties become part of a modern, healthy lifestyle, enhancing Croatia’s winemaking heritage.

This study aimed to determine and compare the antidiabetic potential of the grape skin of old indigenous Croatian white grapevine varieties, grown in the Slavonia and Croatian Danube wine region, through the inhibition effects on AA and AG.

## 2. Material and Methods

### 2.1. Reagents

α-Amylase from porcine pancreas (EC 3.2.1.1, type VI-B; ≥5 units/mg solid), α-glucosidase from *Saccharomyces cerevisiae* (EC 3.2.1.20, type I; 10 U/mg protein), acarbose (pharmaceutical secondary standard), 4-methylumbelliferyl α-D-glucopyranoside (fluorogenic α-glucosidase substrate), Folin–Ciocalteu phenol reagent, 2,2-diphenyl-1-picrylhydrazyl (DPPH), gallic acid, ascorbic acid, iodine, and sodium hydroxide were obtained from Sigma (St. Louis, MO, USA). Soluble potato starch, sodium carbonate, sodium phosphate (monobasic and dibasic), and sodium chloride were obtained from Kemika (Zagreb, Croatia). Calcium chloride, ethanol, and dimethyl sulfoxide (DMSO) were obtained from Gram-mol (Zagreb, Croatia). Potassium iodide and acetic acid were obtained from T.T.T. (Sveta Nedelja, Croatia).

### 2.2. Plant Materials

Grape samples of ten indigenous white grapevine varieties (Table 1) were collected from the experimental vineyard of the Faculty of Agrobiotechnical Sciences Osijek, located in Mandićevac (18°14′50″ E, 45°22′05″ N) in the Slavonia and Croatian Danube wine region, at the moment when the sugar content ceased to increase and a sharp drop in acidity in the must began. All studied varieties were planted in 2020 as part of the national security collection of indigenous grape varieties of the Republic of Croatia, within the framework of the National Program for the Conservation and Sustainable Use of Plant Genetic Resources for Food and Agriculture in the Republic of Croatia. The vineyard is situated on slightly acidic, loess soil with a planting distance of 2.2 × 0.8 m. The training system used is Guyot, with one spur pruned to two buds and a long cane with ten buds. During the growing season, all agricultural practices were conducted using the best practices of the Slavonia and Croatian Danube wine region.

### 2.3. Grape Skin Extraction

For each grape variety, 30 undamaged berries were randomly chosen and divided into three replicates with 10 berries each. The berries were dried with filter paper and weighed. The skins were then carefully separated from the pulp, dried between two sheets of filter paper, weighed, and dried in a laboratory drier at 40 °C for 36 h, when no further significant change in weight was observed. After drying, the skins were weighed, with fresh weight (FW) and dry weight (DW) recorded, and ground manually with quartz sand (thin grain 0.25–0.30 mm particle size).

The extraction process involved mixing dried grape skins with 96% ethanol (*v*/*v*) in polyethylene centrifuge tubes, adhering to a 1:15 solid (DW) to liquid ratio. The ultrasonic-assisted extraction was performed in an ultrasonic bath (Branson M2800 ultrasonic, 40 kHz, Branson Ultrasonics, Brookfield CT, USA) for 15 min at a controlled temperature lower than 40 °C. The resulting suspension was cooled to room temperature and subsequently centrifuged by SIGMA 3–18 K centrifuge (Sigma Laborzentrifugen GmbH, Germany) at 14,000 rpm (20,379 RCF) and 20 °C for 10 min. The procedure was then repeated twice more with the remaining residue. All supernatants were collected in one extract and stored at −20 °C until further analysis.

### 2.4. Determination of Total Phenol Content

The total phenol content of grape skin extracts (GSEs) was determined spectrophotometrically using the Folin–Ciocalteu assay [28,29]. An aliquot of each extract (10 μL) was diluted up to 3 mL with ultra-pure water, followed by 500 μL of freshly diluted 1:1 Folin–Ciocalteau reagent (*v/v*). Subsequently, 2 mL of 10% (*w/v*) Na_2_CO_3_ solution was added and the mixture was left in the dark for 60 min. The absorbance versus negative control (without extracts) was read at 725 nm (S-220 UV/VIS Spectrophotometer, Boeco, Germany). The same procedure was applied for standard solutions of gallic acid (0–5 μg/mL in the reaction mixture). The assay was performed in triplicate for all samples. Total phenols were expressed as mg of gallic acid equivalents per 100 g of grape skin dry weight (mg GAE/100 g DW) using a calibration curve with standard gallic acid.

### 2.5. Determination of Antioxidant Activity

The determination of antioxidant activity (AOA) against the DPPH radical was performed according to the procedures of Von Gadow et al. (1997) [30] and Marinova et al. (2011) [31] and adapted to the 96-well microplate. Each extract (5 μL) was diluted up to 125 μL with ethanol and mixed with 125 μL of 100 μM DPPH in ethanol, to a final volume of 250 μL. The mixtures were shaken and stored in the dark for 30 min at room temperature. The absorbance of the reaction mixture regarding the control (without extracts) was read at 517 nm using a microplate reader (TECAN Spark Multimode Microplate Reader, Tecan Trading AG, Switzerland).

The same procedure was applied for standard ascorbic acid solutions (0–6 μg/mL in the reaction mixture). The assay was performed in triplicate for all samples. Antioxidant activity was expressed as mg of ascorbic acid equivalents per 100 g of grape skin dry weight (mg AAE/100 g DW) using a calibration curve with standard ascorbic acid.

### 2.6. In Vitro α-Amylase Inhibitory Assay

The α-amylase inhibitory assay was measured as the decrease in absorbance of the starch–triiodide complex [32]. The GSE (10 μL) was diluted up to 890 μL with sodium phosphate buffer (0.02 M, with 6 mM NaCl and 1 mM CaCl_2_, pH 6.9), mixed with 10 μL of α-amylase (10 units/mL, in 0.02 M sodium phosphate buffer with 6 mM NaCl, pH 6.9), and preincubated at 37 °C for 15 min. Then, 100 μL of 2% (*w/v*) starch solution was added and the reaction mixture was incubated for 15 min at 37 °C. The reaction was stopped with 1 mM triiodide solution in 1 M acetic acid. The reaction mixture was diluted with 1 mL of ultra-pure water, and the absorbance (A) was immediately read at 620 nm (S-220 UV/VIS Spectrophotometer, Boeco, Germany) against the blank containing all reagents except the enzyme solution. The inhibition assay was performed in triplicate for all samples. The negative control included all reagents except the extract. The positive control was the standard α-amylase inhibitor, acarbose at a concentration of 0.6667 mg/mL (the same as the final concentration of extracts in the reaction mixture).

Inhibition% = A_(negative control)_ − A_(sample)_/A_(negative control)_ × 100% (1)

### 2.7. In Vitro α-Glucosidase Inhibitory Assay

The method for the α-glucosidase inhibitory activity assay [33,34] was adjusted for a 96-well microtiter plate. Briefly, 20 μL of the α-glucosidase solution (0.1 units/mL, in 0.1 M sodium phosphate buffer, pH 6.8) was mixed with 160 μL of diluted extract solution (1 μL of original extract with 159 μL 0.1 M phosphate buffer, pH 6.8). After 15 min of preincubation at 37 °C with constant shaking (PHMT Thermoshaker, Grant Instruments Ltd., Cambridge, UK), 20 μL 4-methylumbelliferyl α-D-glucopyranoside solution (1 mM in DMSO) as the substrate was added to start the reaction. The reaction mixture was incubated at 37 °C for 20 min and terminated by the addition of 100 μL of 0.5 M Na_2_CO_3_ in 0.1 M NaOH. Enzymatic activity was measured as the fluorescence intensity (FI) of the released 4-methylumbelliferone against the blank containing all reagents except the enzyme solution on a Perkin-Elmer Fluorescence Spectrometer (FL 6500, PerkinElmer, Shelton, CT, USA), with excitation and emission set at 360 and 450 nm, respectively. The inhibition assay was performed in triplicate for all samples. The negative control included all reagents except the extract, and for the positive control, acarbose (0.3334 mg/mL, same as the final concentration of GSE in the reaction mixture) was used. Inhibitory activity was calculated using the equation:

Inhibition% = FI_(negative control)_ − FI_(sample)_/FI_(negative control)_ × 100% (2)

### 2.8. Statistical Analysis

All experiments were carried out in triplicate, and the results are expressed as mean ± standard deviation (SD). The results of the total phenols, antioxidant activity, α-amylase, and α-glucosidase inhibitory assay between different grape varieties were compared by analysis of variance (one-way ANOVA). Tukey’s test was used to find significant differences between means at the *p* = 0.05 level. Principal component analysis (PCA) was applied to examine the interrelations among a set of grape varieties according to their inhibitory effects on α-amylase and α-glucosidase. Statistical analyses were performed with Statistica 14 (TIBCO Software Inc., 2020, Palo Alto, CA, USA).

## 3. Results and Discussion

The list of analyzed grape varieties with the data of the average mass of ten berries (AM10B/g), the average mass of fresh skin of ten berries (AMFS10B/g), and the average mass of dry skin of ten berries (AMDS10B/g) is given in Table 1.

### 3.1. Total Phenolic Content and Antioxidant Activity

**Figure 1:**
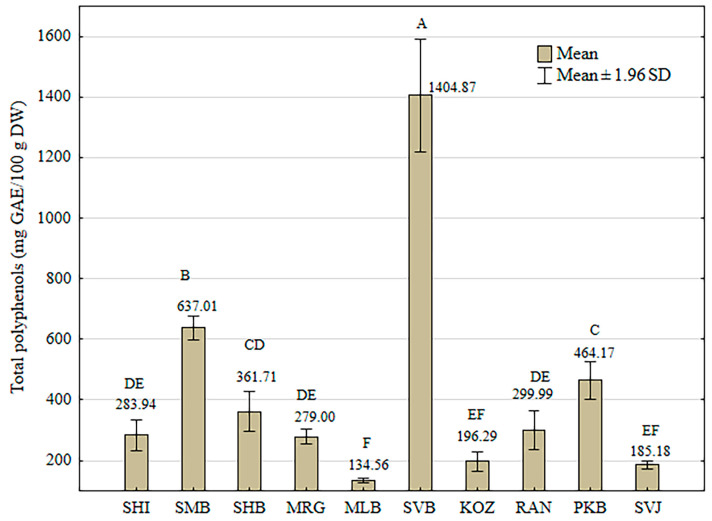
Total polyphenol (TP) content in the grape skin extracts of ten indigenous Croatian white grapevine varieties determined by the Folin–Ciocalteu method, expressed as mg gallic acid equivalents (GAE) per 100 g dry weight (DW) of grape skin. Each value represents the mean ± 1.96 standard deviations of the mean. Different capital letters represent significant differences between varieties at the 0.05 level determined by the Tukey test.

The results of the determination of total phenolic (TP) content in the GSE determined by the Folin–Ciocalteu method are presented in Figure 1.

The GSE of “Svetokriška belina” (SVB) had the highest total phenolic content (1404.87 ± 94.59 mg GAE), which was significantly different from the second-highest TP content of the GSE of “Smudna belina” (SMB) (637.01 ± 19.60 mg GAE). The lowest TP contents were “Kozjak” (KOZ) (196.29 ± 16.14 mg GAE), “Svjetljak” (SVJ) (185.18 ± 6.41 mg GAE), and “Mala belina” (MLB) (134.56 ± 4.28 mg GAE).

The results of the TP content in the white GSE are in good agreement with those reported previously in the literature. Thus, the ethanol extract from white grape skin reached the average TP content of 500 mg GAE/100 g DW of grape skin of the Portuguese white grape variety, Dona Maria [35], or the Serbian white variety, Župljanka (940 mg/100 g DW of grape skin) [36]. In the skin of white grape, the phenolic content is predominantly comprised of flavan-3-ols (catechin, epicatechin, gallocatechin gallate, and catechin-3-gallate), flavonols (quercetin, rutin, and myricetin), flavanons (naringin), flavons (apigenin), coumarins (aesculin), hydroxybenzoic acids, hydroxycinnamic acids [13,14,36,37], resveratrol, and proanthocyanins (condensed tannins) [15]. The phenolic compound profile and contents play important roles in the quality and health effects of grapes. The major groups of compounds identified in organic extracts from grape skins that influence grape flavor and aroma include fatty acids, higher alcohols, sterols, and terpenoids [16].

**Figure 2:**
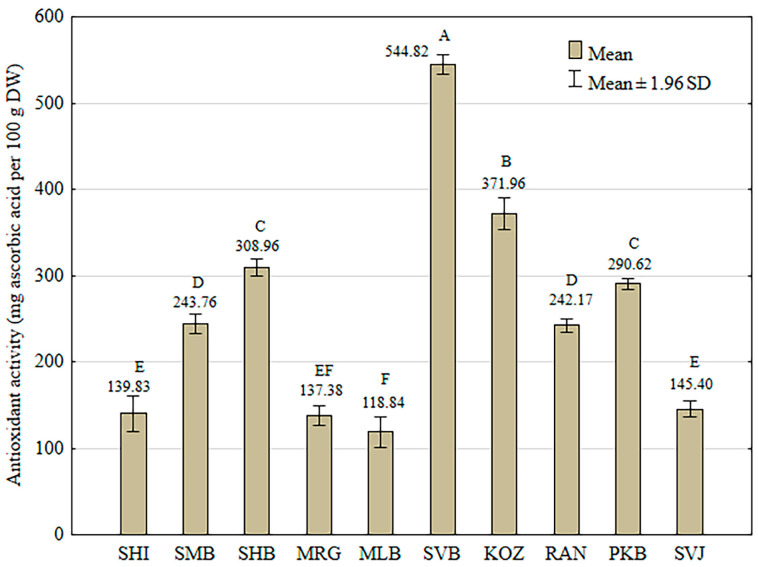
Antioxidant activity against DPPH of the grape skin extracts of ten indigenous Croatian white grapevine varieties. Each value represents the means of three values ± 1.96 standard deviations. Different capital letters represent significant differences between varieties at the 0.05 level determined by Tukey’s test.

Figure 2 presents the results of the antioxidant activity assay against DPPH expressed as mg of ascorbic acid equivalents per 100 g of grape skin dry weight. The highest antioxidant activity was observed in the GSE of “Svetokriška belina” (544.82 ± 6.06 mg AAE), which correlates well with the highest total phenol content of this variety. In contrast, the skin extract of “Kozjak”, a variety with one of the lowest TP contents, exhibited the second highest antioxidant activity. Among the varieties, “Mala belina” had the lowest antioxidant capacity (118.84 ± 8.98 mg AAE) and the lowest TP content as well.

A linear regression analysis confirmed the close relationship between total polyphenol content and antioxidant activity. The linear regression equation that relates AOA with the TP of GSE is:

AOA = 135.32 + 0.28 TP DW(3)

*R* = 0.79; *R*^2^ = 0.62; *F*_1,8_ = 13.08; *p* = 0.007.

**Figure 3:**
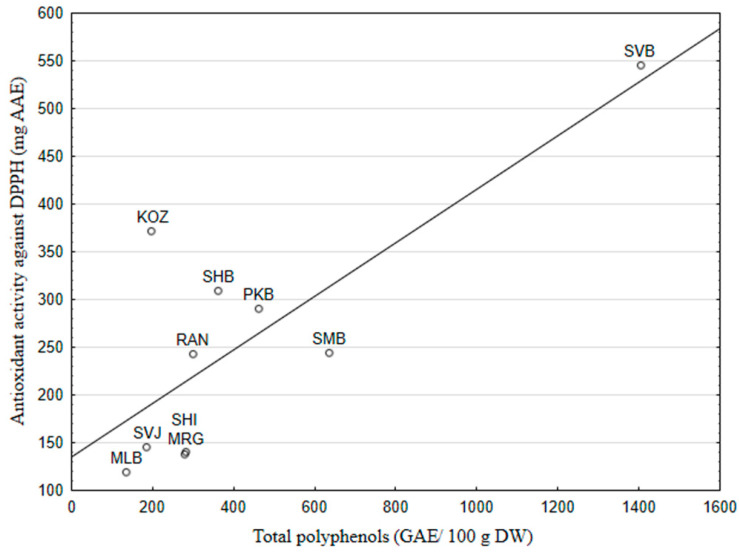
Relationship of antioxidant activity against DPPH and total polyphenols of the grape skin extracts of ten indigenous Croatian white grapevine varieties.

Observed and predicted values of AOA obtained by Equation (1) are shown in Table S1. Figure 3 shows the scatterplot of AOA against the TP of GSE. An inspection of the graph and residuals in Table S1 reveals that the GSE antioxidant activities of the varieties “Svetokriška belina”, “Pikasta belina” (PKB), and “Ranfol” (RAN) were the most related to their total polyphenol content. The positive relationship between the higher total phenolic content and higher antioxidant activity of the grape skin extracts was also evident in the literature [35,38]. Dabetić et al. [36] confirmed a significant correlation of AOA with TP for both seeds and skin extracts (0.798 ≤ *R* ≤ 0.967; *p* < 0.01). A negative relationship between high AOA and low TP for the skin extract of “Kozjak” can be explained by the fact that grapes contain other nutritional antioxidants, such as carotenoids and vitamin C, in addition to phenols. A low correlation was also observed between antioxidant activity and total phenolic content of the white grape variety Muscat blanc [39], and grapes of different varieties from Chile [40]. The low correlations suggest that the grape extract’s ability to interact with reactive species is not solely determined by phenolics. The non-phenolic antioxidant constituents in grapes are carotenoids, and the most important identified in grapes are ß-carotene and lutein. Climate and plant variety determine the profile and carotenoid content of grapes. The study by Bunea et al. [38] found a higher concentration of carotenoids in white grape varieties compared to the blue-black cultivars.

### 3.2. Inhibition of α-Amylase and α-Glucosidase by Grape Skins Extracts

**Figure 4:**
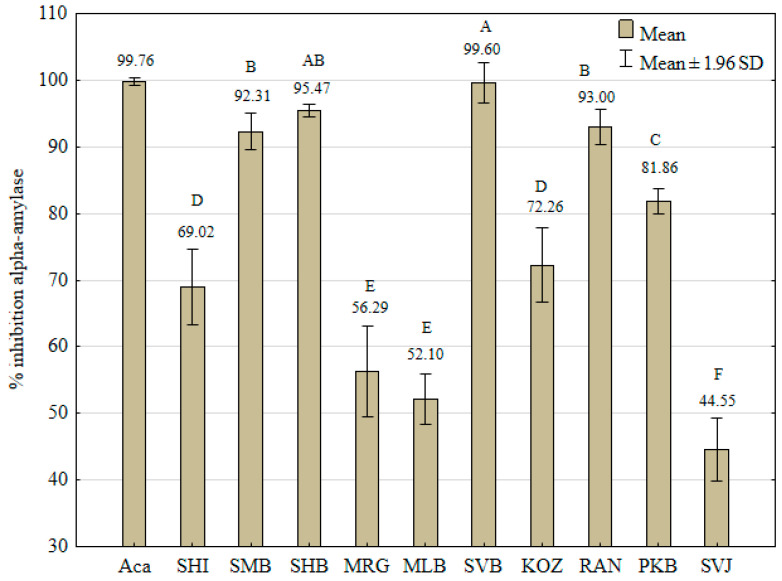
Inhibition of α-amylase grape skin extracts of ten indigenous Croatian white grapevine varieties, expressed as % inhibition according to the positive control acarbose (Aca) (0.6667 mg/mL). Each value represents the means of three values ± 1.96 standard deviations. Different capital letters represent significant differences between varieties at the 0.05 level determined by the Tukey test.

The results of the in vitro α-amylase inhibition assay are depicted in Figure 4. The standard inhibitor, acarbose, displayed mean % inhibition (99.76 ± 0.30) at the concentration of 0.6667 mg/mL, which corresponds to the final concentration of grape skins’ DW in the reaction mixture.

The GSE of “Svetokriška belina” showed the highest inhibitory activity (99.60 ± 1.51%), which was significantly similar only to “Šemnička belina“ (SHB, 95.47 ± 0.50). “Šemnička belina“ had significantly similar means of inhibition to “Ranfol” (93.00 ± 1.36%) and “Smudna belina” (92.31 ± 1.42%). “Svetokriška belina” showed the expected high inhibition against α-amylase due to its high TP content and AOA. “Smudna belina” had the second highest TP, but it had a medium AOA potential, while “Ranfol” had a low TP and medium AOA. Regression analysis showed a low linear correlation between the inhibition of α-amylase and TP (*R* = 0.64; *R*^2^ = 0.41; *F*_1,8_ = 5.51; *p* = 0.05). A closer relationship was confirmed between amylase inhibition and AOA potential (*R* = 0.72; *R*^2^ = 0.52; *F*_1,8_ = 8.54; *p* = 0.02).

It may be concluded that GSEs’ anti-amylase abilities are related to their polyphenol components, as well as to their other antioxidants, such as terpenes [41,42] and carotenoids [38]. Also, differences in the α-amylase inhibition could be attributed to the differences in the content of individual polyphenols. For example, previous studies have demonstrated that the inhibitory potential against α-amylase increased with the increasing mean degree of proanthocyanidin polymerization [43,44].

**Figure 5:**
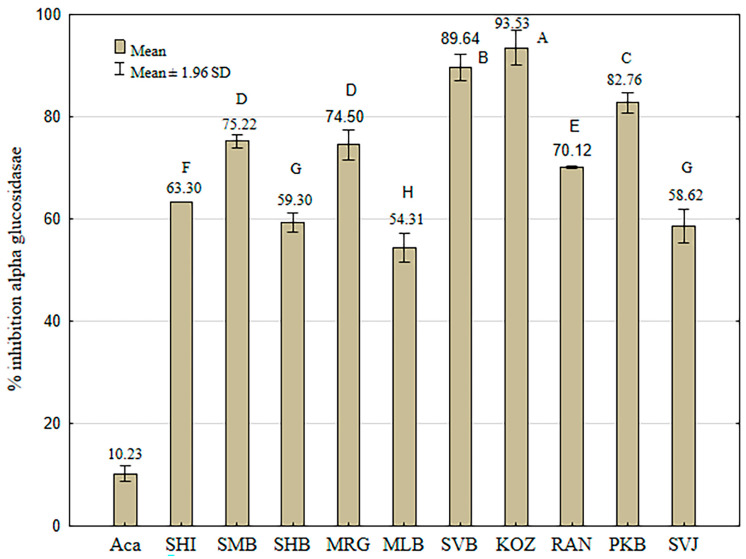
Inhibition of α-glucosidase grape skin extracts of ten indigenous Croatian white grapevine varieties, expressed as % inhibition according to the positive control acarbose (0.3334 mg/mL). Each value represents the means of three values ± 1.96 standard deviations. Different capital letters represent significant differences between varieties at the 0.05 level determined by the Tukey test.

The standard inhibitor, acarbose, showed lower inhibition potential against α-glucosidase than against α-amylase at the same concentration, 0.6667 mg/mL (37.82 ± 0.84%), which is consistent with previous results [24,45]. The standard inhibitor, acarbose, exhibited the mean % inhibition (10.23 ± 0.82) at the concentration of 0.3334 mg/mL, which corresponds to the final concentration of extracts in the reaction mixture. Generally, GSE showed a more pronounced inhibitory effect on α-glucosidase compared with α-amylase; therefore, the final concentration of grape skins’ DW in the reaction mixture was 0.3334 mg/mL, twice lower than for the α-amylase inhibition assay. These results confirm the study by Campos et al. [24], which demonstrated higher inhibitory activity of polyphenols and polysaccharide–polyphenol conjugates extracted from grape pomace against α-glucosidase than α-amylase. The results of the α-glucosidase inhibition assay of individual GSE extracts of different varieties are presented in Figure 5.

The highest inhibition potential was exhibited by the GSE of “Kozjak” (93.53 ± 1.73%). A significantly lower value than “Kozjak” was shown by the GSE of “Svetokriška belina” (89.64 ± 1.32%), followed by the GSE of “Pikasta belina” (82.76 ± 1.01%). The lowest values of inhibition against α-glucosidase were demonstrated by the GSE of Svjetljak” (58.62 ± 1.68%) and “Mala belina” (54.31 ± 1.45%), similar to the inhibition of α-amylase.

A significant correlation of the inhibition of α-glucoside was confirmed only with the antioxidant activities of GSEs (*R* = 0.74; *R*^2^ = 0.54; *F*_1,8_ = 9.41; *p* = 0.02).

Probably, the variability in the inhibitory potential of an individual GSE against α-glucosidase can be attributed to the difference in polyphenol content. It is proven that some polyphenols have shown a high affinity towards α-glucosidase active sites, such as free polyphenols, phenolic acids, and polymerized pigments, because of strong interactions with the active site of the enzyme [24,46]. The GSE of “Kozjak” was shown as an excellent source of natural non-phenolic antioxidants and exhibited the highest inhibition potential against α-glucosidase. Besides polyphenols, important antioxidants in grapes are carotenoids, whose capacity was proven for pure compounds and in grape extracts. Besides ß-carotene and lutein, other carotenoids are present in grapes: neochrome, neoxanthin, violaxanthin, luteoxanthin, flavoxanthin, and zeaxanthin [47,48]. Carotenoids isolated from different algae and plants have shown inhibitory activity against α-glucosidase [49,50,51,52]. Along with carotenoids, other important constituents of grape skin, terpenoids, are proven inhibitors of α-glucosidase [42,53,54]. In Croatia, it has been determined that the variety “Kozjak” is the parent of one variety from the “belina” group (“Belina Hižakovo”), and in the second generation it is related to “Šemnička belina”. Given that as a wine variety, it produces wines of mediocre quality, the variety is critically endangered due to its small population, but especially because its characteristics are currently not interesting for commercial production [55].

**Figure 6:**
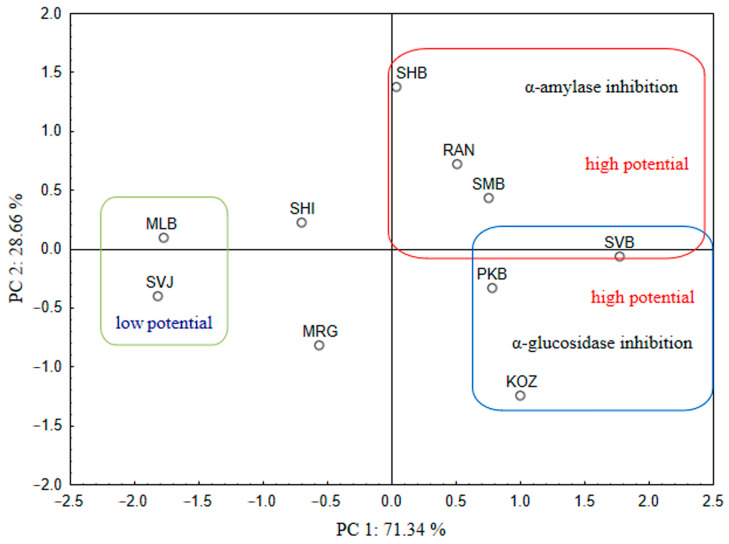
Two-dimensional principal component analysis plot of α-amylase and α-glucosidase inhibition potential of the grape skin extracts of ten indigenous Croatian white grapevine varieties.

To classify grape varieties according to their ability to inhibit α-amylase and α-glucosidase, principal component analysis (PCA) was used. The PCA results are shown as a plot representing a projection of the grape varieties on the factor plane (Figure 6). The first principal component (PC 1) explained 71.34% of the variance, and PC 2 28.66% of the variance. The PCA plots show the most potent varieties as being located on the positive side of both PCs, while the least active variety, “Svjetljak”, is seen on the diagonal opposite edge. The most potent varieties against α-glucosidase are located in the lower right part of the PCA plot, while the variety “Mala belina” is on the opposite side on the upper left side of the PCA plot.

## 4. Conclusions

This study proved the potential antidiabetic effect of grape skin extracts from indigenous Croatian white grapevine varieties. As the most potent grape variety, “Svetokriška belina” stands out against the enzymes α-amylase and α-glucosidase. The “Kozjak” variety was second best, with good inhibitory potential for α-glucosidase. The inhibition abilities of GSEs are related to their antioxidant activities, and only partially to their total phenolic content. Therefore, in further studies, it is necessary to perform a comprehensive qualitative and quantitative analysis of skin extracts of the leading grape varieties. The research results will enable us to provide an outline for the further design of possible natural supplements for the prevention and treatment of diabetes. This could encourage the economic revitalization of indigenous Croatian grapevine varieties.

## Figures and Tables

**Table 1:** List of analyzed grape varieties with data of mean values (±SD) of the mass of ten berries (AM10B/g), the mass of fresh skin of ten berries (AMFS10B/g), and the mass of dry skin of ten berries.

Grape Variety	Grape Code	AM10B/g	AMFS10B/g	AMDS10B/g
Šipelj	SHI	19.88 ± 0.39	2.68 ± 0.41	0.83 ± 0.09
Smudna belina	SMB	13.40 ± 0.39	1.86 ± 0.14	0.56 ± 0.04
Šemnička belina	SHB	22.38 ± 0.22	3.84 ± 0.27	1.34 ± 0.11
Mirkovačka belina	MRB	18.74 ± 2.06	1.52 ± 0.14	0.46 ± 0.04
Mala belina	MLB	17.40 ± 0.37	2.19 ± 0.18	0.65 ± 0.05
Svetokriška belina	SVB	15.07 ± 0.90	1.94 ± 0.16	0.66 ± 0.06
Kozjak	KOZ	24.86 ± 0.71	3.24 ± 0.37	1.09 ± 0.10
Ranfol	RAN	16.56 ± 0.36	2.10 ± 0.17	0.62 ± 0.03
Pikasta belina	PKB	22.00 ± 1.06	2.78 ± 0.04	0.80 ± 0.01
Svjetljak	SVJ	17.30 ± 0.79	2.81 ± 0.16	0.83 ± 0.04

## Data Availability

The original contributions presented in this study are included in the article/Supplementary Materials. Further inquiries can be directed to the corresponding author.

## Supplementary Materials

The following supporting information can be downloaded at: https://www.mdpi.com/article/10.3390/foods13244143/s1, Table S1. Observed and predicted value of antioxidant activity against DPPH (mg AAE/100 g DW) of the grape skin extracts calculated by equation: AOA = 135.32 + 0.28 TP DW.
