# Supplementary material for: The Antidiabetic Effect of Grape Skin Extracts of Selected Indigenous Croatian White Grapevine Varieties
Source: Foods. 2024 Dec 20;13(24):4143. doi: 10.3390/foods13244143 (PMC11675538; doi:10.3390/foods13244143)
Supplement: Supplementary file 1 [file foods-13-04143-s001.zip › foods-3367748-supplementary.pdf]

Table S1. Observed and predicted value of antioxidant activity against DPPH (mg AAE/100 g DW) of the grape skin extracts calculated by equation: AOA = 135.32 + 0.28 TP DW.

	Observed Value	Predicted Value	Residual
SHI	139.83	214.92	-75.10
SMB	243.76	313.90	-70.14
SHB	308.96	236.72	72.23
MRG	137.38	213.54	-76.16
MLB	118.84	173.05	-54.20
SVB	544.82	529.15	15.66
KOZ	371.96	190.35	181.61
RAN	242.17	219.42	22.75
PKB	290.62	265.45	25.17
SVJ	145.40	187.24	-41.84
